# Comparison of Embedded Sensor Data for Long-Term Care Residents Before and After Onset of the COVID-19 Pandemic

**DOI:** 10.1093/geroni/igab046.360

**Published:** 2021-12-17

**Authors:** Erin Robinson, Wenlong Wu, Geunhye Park, Gashaye M Tefera, Kari Lane, Marjorie Skubic, James Keller, Mihail Popescu

**Affiliations:** University of Missouri, Columbia, Missouri, United States

## Abstract

Older adults have experienced greater isolation and mental health concerns during the COVID-19 pandemic. In long-term care (LTC) settings, residents have been particularly impacted due to strict lockdown policies. Little is known about how these policies have impacted older adults. This study leveraged existing research with embedded sensors installed in LTC settings, and analyzed sensor data of residents (N=30) two months pre/post the onset of the U.S. COVID-19 pandemic (1/13/20 to 3/13/20, 03/14/20 to 5/13/20). Data from three sensors (bed sensors, depth sensors, and motion sensors) were analyzed for each resident using paired t-tests, which generated information on the resident’s pulse, respiration, sleep, gait, and motion in entering/exiting their front door, living rooms, bedrooms, and bathrooms. A 14.4% decrease was observed in front door motion in the two months post-onset of the pandemic, as well as a 2.4% increase in average nighttime respiration, and a 7.6% increase in nighttime bed restlessness. Over half of our sample (68%) had significant differences (p<0.05) in restlessness. These results highlight the potential impact of the COVID-19 pandemic and social distancing policies on older adults living in LTC. While it is not surprising that significant differences were found in the front door motion sensor, the bed sensor data can potentially shed light on how sleep was impacted during this time. As older adults experienced additional mental health concerns during this time, their normal sleep patterns could have been affected. Implications could help inform LTC staff, healthcare providers, and self-management of health approaches among older adults.

